# Vasculitides and occluding vasculopathies, challenges in recognizing histopathological patterns, and their solutions

**DOI:** 10.3389/fmed.2022.994450

**Published:** 2023-02-01

**Authors:** Michael Wilk, Bettina G. Zelger, Bernhard Zelger

**Affiliations:** ^1^Private Dermatohistological Laboratory, Nuremberg, Germany; ^2^Institute of Pathology, Medical University Innsbruck, Innsbruck, Austria; ^3^Private Dermatohistopathological Laboratory Zelger, Innsbruck, Austria

**Keywords:** cutaneous vasculitis, vasculitis, occluding vasculopathy, coagulopathy, algorithm, review

## Abstract

In this review, we propose a classification of vasculitides and occluding vasculopathies using the clinicopathological correlation as the basic process. We use an algorithmic approach with pattern analysis, which allows reliable reporting of microscopic findings. We first differentiate between small and medium vessel vasculitis. Second, we differentiate the subtypes of small- and medium-sized vessels. Finally, we differentiate vasculitides according to the predominant cell type into leukocytoclastic and/or granulomatous vasculitis. Regarding leukocytoclastic vasculitis as a central reaction pattern of cutaneous small/medium vessel vasculitides, its relation or variations may be arranged in a wheel-like order. With respect to occluding vasculopathies, the first two steps are identical to the algorithm of vasculitides, and we finally differentiate according to the time point of the coagulation/reorganization process and the involved inflammatory cells/stromal features. By visualizing the criteria in the style of bar codes, clinical and histological overlaps and differences may become more transparent.

## 1. Introduction

Vasculopathies include inflammatory, neoplastic, and genetic vascular disorders. *Vasculitis* (1–15) must be differentiated from *occluding vasculo-including coagulopathies*. The former is defined as an increased number of inflammatory cells in and/or around a vessel wall accompanied by vascular damage. This may be signified by leukocytoclasia, endothelial, and smooth muscle necrosis as well as fibrin deposition and thrombi, or connective tissue degeneration. Fibrin may not always be evident but if present it serves as a clue to the diagnosis (14). Occluding vasculopathies/coagulopathies (16–18) are defined as vascular disorders with partial or complete occlusion of one or more vessels caused by hypercoagulability, especially thrombi and emboli. They can be seen in the context of malignant proliferations, malformations, hamartomas, or genetic disorders such as neurofibromatosis, embolization of different materials (cholesterol, oxalate, and microorganisms), or most commonly by coagulopathies. In contrast to vasculitides occluding vasculopathies are initiated by abnormal blood coagulation. This distinction has important therapeutic consequences. However, the two processes may be intertwined. Vasculitis can trigger vaso-occlusive events, as in septic vasculitis, and occluding vasculopathies may mimic vasculitis since in later stages they show a secondary inflammatory infiltrate composed especially of lymphocytes. When coexisting coagulation abnormalities exacerbate vasculitic damage, disastrous disease courses may occur (10, 11, 16–20).

Our approach uses the clinicopathological correlation as the main process for classification (12, 17, 18). We use an algorithmic approach with pattern analysis, which facilitates the interpretation of microscopic findings. Thus, similar to the International Chapel Hill Consensus Conference on the nomenclature of vasculitis and its dermatologic addendum published recently (21–23), we *first* differentiate between small and medium vessel vasculitis (large vessels do not exist in the skin). “Small vessels” comprise capillaries and postcapillary venules in contrast to “medium vessels” when primarily arteries (for simplicity also arterioles) or veins at the border of the reticular dermis/subcutis are involved. In the *second* step, the subtypes of small- (capillaries vs. postcapillary venules) and medium-sized (arterioles/arteries vs. veins) vessels are to be determined. Capillaries are tiny vessels that are not only present in dermal papillae, in perifollicular and periglandular connective tissue but also between collagen fibers in the reticular dermis, and within the lobules of the panniculus; in the latter, the blood supply is terminal (24–26). In contrast, postcapillary venules are seen at the border between the papillary and reticular dermis and also at the border between the reticular dermis and subcutis, and thus contribute to the superficial and deep vascular plexus. Interconnecting venules between the superficial and deep plexus and septal venules are present as well. In addition, arteries must be differentiated from veins by criteria such as texture of smooth muscle layers or the “artery cross sign” (12, 27). As a characteristic sign and clue of inflammatory and thrombotic vascular disease, both arteries and veins show a prominent corona of vasa vasorum (12). This phenomenon can be seen not only in the early stages of the disease but also later when all other inflammatory features are gone, and the previous vessel has shrunken to a small, obliterated strand of collagen tissue. In the *final* step, we differentiate the vascular process into leukocytoclastic and/or granulomatous vasculitis. Thus, leukocytes and their debris are most typical for early authentic vasculitis, and macrophages present in different forms of granulomas are typical of vasculitis associated with a significant connective tissue alteration. Furthermore, extravascular histopathological findings such as tissue eosinophilia (eosinophilic granulomatosis with polyangiitis, EGPA), blue/red collagenolytic granulomas (granulomatosis with polyangiitis, GPA, and EGPA), or storiform fibrosis (granuloma faciale) may serve as a hint to the correct diagnosis. However, these clinicopathological findings represent reaction patterns caused by a variety of etiologic factors or pathogenetic mechanisms. Moreover, there are patients whose clinicopathologic presentation changes over time making clinical follow-up mandatory.

**FIGURE 1:**
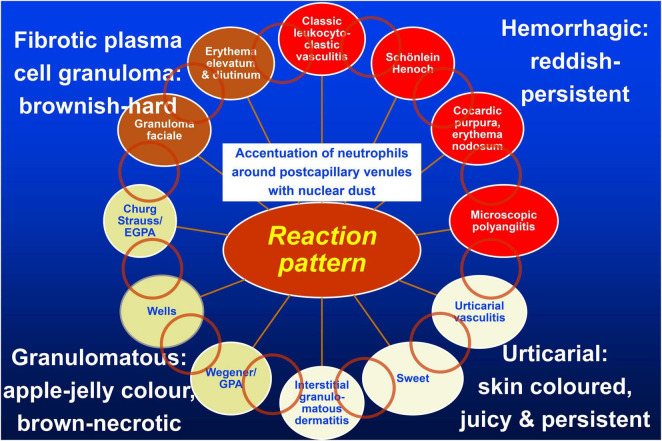
Vasculitis wheel: Leukocytoclastic vasculitis with the accentuation of neutrophils around postcapillary venules and nuclear dust is the connecting histopathological finding. The clinical presentations of classic leukocytoclastic vasculitis, Schönlein Henoch purpura, cocardic purpura, and microscopic polyangiitis are typically *hemorrhagic and thus reddish and persistent* clinically and on diascopy; urticarial vasculitis, Sweet’s syndrome, and interstitial granulomatous dermatitis are *urticarial and thus skin-colored, edematous, with only subtle hemorrhage, and no yellow/apple-gelee color* on diascopy; GPA, Wells’syndrome, and EGPA are *granulomatous and brown, with a yellow/apple-gelee color* on diascopy. Necrotic lesions with erosions and ulcers may occur. Finally, granuloma faciale and erythema elevatum diutinum present as *firm brown nodules*. The light brown circles indicate individual clinical and histopathological overlap between the diseases. Published with permission from JDDG, Wiley.

Our approach to occluding vasculopathies is similar with variation of the *final* step, in which we differentiate according to the life cycle of the event (17, 18). In the early stages, fibrin thrombi are dominant which can affect capillaries, postcapillary venules, or larger vessels, especially when the intensity of the process increases. At this early stage, prominent hemorrhage is a frequent finding, usually presenting as non-inflammatory retiform purpura, likely due to ischemia and the leakage of vessels prior to complete occlusion. According to the extent and duration of the disease, clinically necrotic lesions with erosions to ulceration, cellular debris with crust formation are evident, and histopathologically granulomatous tissue with a mixed infiltrate of neutrophils, lymphocytes, and macrophages. In due course, the degradation of these thrombi leads to “lymphocytic vascular reorganization” (17, 18), a process dominated by a dense perivascular lymphocytic infiltrate that more surrounds than invades the walls of affected vessels. This histopathological process caused by occluding vasculopathies has been referred to as “secondary vasculitis” (1–4). As it is not vasculitis, we recommend that this term should be avoided. Finally, there is healing with the complete reconstitution of vessels with lumina, and/or partial to complete occlusion by fibroblasts and collagen. Deep erosions (in particular ulcers) heal with scars, as seen in atrophie blanche. This life cycle of histopathological events in occluding vasculopathies is seen in a variety of instances. Any erosion, ulceration, or necrotic lesion causes hypercoagulopathy with fibrin thrombi in the vessels of the surrounding tissue. The clue to differentiate from vasculitis is the distribution of the inflammation. This process is accentuated in the vicinity of the erosion/ulcer in the case of occluding vasculopathies and decreases gradually with increasing distance, while vasculitis will focus on dermal and/or subcutaneous vessels. Nuclear dust is present in both conditions and does not help in the differential. The final diagnosis of various causes of occluding vasculopathies must be done by clinicopathological correlation. The same applies to vasculitides illustrated by the “vasculitic wheel” demonstrating various clinical presentations linked by the reaction pattern of leukocytoclastic vasculitis [Figure 1; (12)]. In the following, we present a classification of small- and medium-sized vasculitis based on the histopathological criteria stressing the least common denominator and additional characteristic findings (12). This section is followed by an algorithmic approach of occluding vasculopathies and coagulopathies [Table 1; (17, 18)]. The primary histological investigation must be supplemented by appropriate clinical work- and follow-up of the individual patient.

**TABLE 1:** Vasculo-/coagulopathies—systematic approach and clinicopathological comparison reminiscent of a bar code-reader.

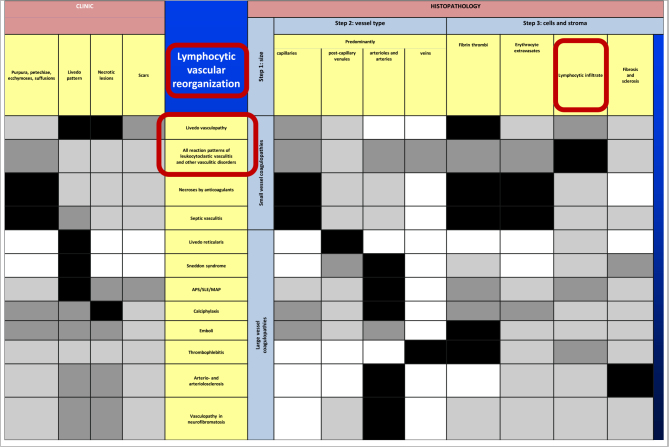

All inflammatory and even proliferative/neoplastic processes may be associated with coagulation disorders and thus, with fibrin thrombi; this can lead to lymphocytic vascular reorganization visualized and emphasized by red circles.

APS, antiphospholipid syndrome; SLE, systemic lupus erythematosus; MAP, malignant atrophic papulosis. Published with permission from JDDG, Wiley.

## 2. Subsections relevant to the subject

### 2.1. Small vessel vasculitis

The different variations of leukocytoclastic vasculitis belong to the spectrum of cutaneous small vessel vasculitis. Manifestations generally accepted to belong to this entity are classic leukocytoclastic vasculitis and its variants like immune complex (IgG/IgM) small vessel vasculitis, IgA small vessel vasculitis/Schönlein Henoch (28, 29), cocardic purpura Seidlmeyer Finkelstein (30) as well as more localized forms such as granuloma faciale and erythema elevatum diutinum. In contrast, the association between leukocytoclastic vasculitis and Sweet’s syndrome (31), Wells’ syndrome (32), and interstitial granulomatosis dermatitis is a matter of controversy. In our understanding, these are variants of leukocytoclastic vasculitis as well and are therefore discussed in this framework. In addition, we recently reported on a series of patients with early erythema nodosum histologically showing the features of leukocytoclastic vasculitis affecting the postcapillary venules in the septa of the subcutaneous fat (26). Since Sweet’s syndrome may rarely present in subcutaneous location as well there is an obvious histopathological overlap between these entities. All of them reveal an inflammatory process *accentuated around postcapillary venules and perivascular leukocytoclasia* as the least common denominator. Additional characteristic histopathological findings are inserted in the following list of diseases. Of note, all these processes tend to spare or at least affect to a lesser extent the capillaries than the postcapillary venules (12).

1. Classic leukocytoclastic vasculitis
  – Fibrin within/around vessel walls
  – Perivascular erythrocyte extravasation
2. Granuloma faciale and erythema elevatum diutinum
  – Variable eosinophils
  – Plasma cells and fibrosclerosis
3. Sweet’s syndrome
  – Edema in the papillary dermis
4. Wells’ syndrome
  – Eosinophils with flame figures and palisading macrophages
5. Interstitial granulomatous dermatitis
  – Collagen degeneration with mild basophilic necrotic lesions surrounded by palisading macrophages (granulomas)

### 2.2. Small and medium vessel vasculitis

The vasculitis of small- and medium-sized vessels may occur simultaneously or subsequently (12, 33). Macules, patches, papules, and plaques may be the clinical presentation in small vessel disease, whereas nodules and tumors become visible in medium vessel involvement (34). Other organs may be involved as well which may precede, occur simultaneously with, or follow the skin lesions (35). All of these processes show an inflammatory process *accentuated around postcapillary venules and larger vessels in the skin and subcutis as well as perivascular and intramural leukocytoclasia* as the least common denominators. Additional important histopathological features are mentioned in the following list of diseases. Further criteria such as endothelial cell damage, fibrin within/around vessel walls, perivascular erythrocyte extravasation, edema of the papillary dermis, the presence of eosinophils or plasma cells and fibrosclerosis, and finally lymphocytic vascular reorganization (“lymphocytic vasculitis”) which should be evaluated as well are variable findings only (12). “Lymphocytic vasculitis” is by some authors considered to represent a distinctive form of vasculitis (1–4), but in our view is a regenerative/secondary form of lymphocytic inflammation which can occur following the various forms of vasculitides (12) and vasculo-/coagulopathies as well (17, 18). Thus, the understanding of vasculopathies continues to evolve, recently also nicely summarized for eosinophilic granulomatosis with polyangiitis (36). In addition, microscopic polyangiitis (MPA) frequently involves internal organs and only rarely affects the skin. When skin manifestation is present, it is clinically highly distinctive in showing besides variable palpable purpura a generalized presentation of erythema nodosum-like lesions of arterioles/arteries and/or postcapillary venules affecting also the trunk and arms. In contrast to MPA, however, early erythema nodosum affects only postcapillary venules of the subcutaneous fat and clinically accentuates the lower legs/extremities (26).

1. Granulomatosis with polyangiitis (GPA, Wegener’s granulomatosis)
  – Collagen degeneration with variable basophilic necrotic lesions surrounded by palisading macrophages (granulomas)
2. Eosinophilic granulomatosis with polyangiitis (EGPA, Churg Strauss syndrome)
  – Collagen degeneration with variable eosinophilic necrotic lesions surrounded by palisading macrophages (granulomas)
  – Prominent eosinophils
3. Microscopic polyangiitis
  – Pathology confined to arterioles/arteries and/or postcapillary venules
  – No granulomas

### 2.3. Medium vessel vasculitis

The inflammatory processes of medium-sized vessels (12, 37) may present as those with a prominent *intramural vasculitis* or a prominent *extravascular/interstitial/soft tissue component*. In the former, significant involvement of the surrounding connective tissue is lacking. In the latter, vasculitis may be missed histologically, but necrotic tissue is prominent in the dermis and more commonly subcutaneous tissue, similar to cases with the medium vessel variants of GPA and EGPA. Clinically, lesions are present as nodules and tumors. Apart from the criteria listed in the following text, there are further ones which should be evaluated such as endothelial cell damage, fibrin within/around vessel walls, perivascular erythrocyte extravasation, edema in the papillary dermis, the presence of eosinophils, plasma cells and fibrosclerosis, and lymphocytic vascular reorganization as described in section 2.1 and 2.2. These are variable findings only and, therefore, of minor importance (12).

1. Polyarteritis nodosa (PAN)
  – Affects arterioles and arteries in the subcutis and at the dermis–subcutis junction
  – Early perivascular, intramural, and intraluminal leukocytoclasia, later lymphocytes and macrophages
  – Pathology confined to vessel site, no extravascular/interstitial/soft tissue granulomas
2. Giant cell arteritis
  – Affects arteries of the subcutis, muscles, or deeper tissues
  – Characteristic giant cells, most prominently close to the internal elastic membrane
  – Pathology confined to vessel site, no extravascular/interstitial/soft tissue granulomas
3. Nodular vasculitis/erythema induratum Bazin
  – Affects arteries (and veins) of the subcutis, muscles, or deep tissues
  – Early perivascular, intramural, and intraluminal leukocytoclasia, later lymphocytes and macrophages
  – Lobular panniculitis with caseating necrotic tissues surrounded by palisading (foamy) macrophages and giant cells, Langhans type, tuberculous granulomas
4. Crohn’s disease
  – Vasculitis affecting arteries and veins of the dermis and subcutis accompanied by leukocytoclasia and non-caseating granulomas with prominent macrophages and giant cells, mostly foreign body type

There are many other medium/large vessel vasculitides including *Takayasu arteritis, Kawasaki syndrome, Behcet*’*s disease, vascular collagen disease of lupus erythematosus, dermatomyositis, Sharp*’*s syndrome, or rheumatoid arthritis*. The vessels in the subcutis and/or deeper soft tissues are involved in these diseases, none is exclusively cutaneous. The inflammatory process of affected arteries in deep tissues causes a vascular occlusion with subsequent ischemia. Alternatively, aneurysms may develop and become the focus of thrombi and emboli (12).

In the following, occluding vasculopathies are covered and are again primarily classified based on the size and nature of the involved vessel. These occluding vasculopathies and coagulopathies are more readily classified than vasculitides, according to their clinical and histological characteristics. In our following algorithmic approach, we, therefore, summarize them in Table 1. We mark the least common denominators in black, the characteristic prominent findings in dark gray, the variable findings in light gray, and the missing criteria are indicated in white. The clinical and histopathological characteristics of the following diseases are summarized in this table (17, 18, 38–40).

### 2.4. Small vessel occluding vasculopathies

1. Livedo vasculopathy
2. Lymphocytic vascular reorganization (“lymphocytic vasculitis”)
3. Necrosis induced by anticoagulants
4. Septic vasculitis

### 2.5. Medium vessel occluding vasculopathies

1. Livedo reticularis
2. Sneddon syndrome
3. Anti-phospholipid syndrome: idiopathic or in association with systemic lupus erythematosus (Hughes-syndrome); malignant atrophic papulosis
4. Calciphylaxis
5. Emboli
6. Thrombophlebitis
7. Arteriosclerosis and arteriolosclerosis
8. Vasculopathy in neurofibromatosis

## 3. Discussion

Previous classifications of vascular disorders suffer from impediments (3–11, 19–22). First, classifications may follow different principles in which clinicopathologic findings, vessel size, etiology, pathogenesis, prognosis, or therapeutic options are considered and, in the past, have frequently been mixed (12). Second, some authors do not clearly distinguish between primary vasculitis and coagulopathy which has important therapeutic consequences (41, 42); classical vasculitides are treated with the immunosuppressive agents; microorganism-associated vasculitides need specific antimicrobial therapy; and coagulopathies require anticoagulants. Third, vasculitides can present a relatively benign single-organ (e.g., cutaneous) disease or systemic disease with an impaired prognosis (13, 21–23, 27, 43, 44). The different tissues from organ to organ lead to varying pathologic processes (e.g., hemorrhage is a common manifestation in the affected loose tissue of the lung; or the accumulation of capillary loops makes glomeruli a predisposed area for vascular disease, most commonly seen in leukocytoclastic vasculitis) impeding a precise comparison of morphologic findings (12). Fourth, subtle changes are readily recognized in the skin and may lead to CNS symptoms, but are asymptomatic in other organs, as for example seen in patients with Sneddon syndrome (38, 39).

The present review clearly separates vasculitides from occluding vasculopathies. Our approach focuses on the clinical and histopathological criteria only. Thus, apart from the histological parameters which are helpful when one has to investigate a histological slide, the clinical presentation aids in the differential diagnosis. We think that starting from leukocytoclastic vasculitis as a central reaction pattern of the cutaneous small/medium vessel vasculitides, its clinical relations or variations may be arranged around it in a wheel-like order [(12), Figure 1]. Hence, there are not only important histological but also clinical overlaps between the different forms of small and small-/medium-sized cutaneous vasculitis. This may lead to a better understanding of these diseases in which clinical follow-up is mandatory. Distinguishing entities in the spectrum of small and medium vessel occluding vasculopathies can benefit from this algorithmic approach as well (17, 18). In this context, it must be realized that there is again no sharp border between small and medium vessels occluding vasculopathies but a continuum. In Table 1, we have used bar codes to stress individually typical clinical and histological features of the different diseases. Their use may simplify the results but can assist in making the different entities more understandable and comparable. In addition, we are aware that this method of classifying vasculopathies has its limits until clear molecular tools are discovered (17, 18).

## Author contributions

All authors listed have made a substantial, direct, and intellectual contribution to the work, and approved it for publication.
